# On the Nature of Tetanus Caused by Strychnine

**Published:** 1847-09

**Authors:** Hermann Meyer

**Affiliations:** Zurich


*On the Nature of Tetanus caused by Strychnine.* By Professor HERMANN MEYER, Zurich.—The tetanic convulsions, which follow the exhibition of Strychnine, are well known; as also the fact that these convulsions can be readily excited as reflex movements. The general use of strychnine in experiments on the nervous system is very much based on the knowledge of these facts, and invites to a discussion of the question as to the mode in which the tetanus originates. The following experiments were instituted for this purpose.

Exp. I. The crural nerve of a frog, suffering from convulsions induced by strychnine, was divided; these immediately ceased in the limb.

Exp. II. No convulsions took place in a limb, in which the nerve had been divided previous to the exhibition of the poison.

From these well known experiments, it follows that the originating cause of the convulsions must be sought for in the central parts of the nervous system. But the question ensues, does the tetanus originate from an affection of an individual portion of the central organ, or in an affection of every individual portion? The following experiments, also somewhat well known, tend to throw some light on the subject.

Exp. III. The brain was removed from a frog; tetanus ensued in the trunk.

Exp. IV. Brain and cerebellum removed, result as in the preceding.

Exp. V. Medulla oblongata removed; same result.

Exp. VI. Medulla oblongata divided; general tetanus ensued.

Exp. VII. The spinal marrow was removed posterior to the giving off of the nerves to the anterior extremities; result as in the preceding case.

From these experiments, it follows that the origin of the tetanus is to be sought for in some cause common to all the central organs of the nervous system, The following experiments show what this cause is.

Exp. VIII. The posterior column of the entire spinal marrow was removed from a frog by means of a fine pair of scissors. A solution of strychnine was then either exhibited by the mouth, or applied directly to the spinal cord. Convulsions occurred only in the jaws and eyes.

Exp. IX. The posterior column of that part of the cord giving off nerves to the posterior extremities was removed. Tetanus occurred only in the head and anterior extremities.

Exp. X. The same portion of cord giving off nerves to the anterior extremities was removed. Tetanus occurred only in the head and posterior extremities.

From these experiments, the author thinks himself entitled to conclude, that the presence of the motor nerves, even though uninjured, does not suffice to produce tetanus; but that there must also be an uninterrupted connexion of the sensory and motor fibres within the central part of the nervous system. The only motions, however, which we recognize as dependent on the sensory fibres, are those which we know to be the cause of reflex movements. The above experiments, therefore, would lead to the conclusion, that tetanus caused by strychnine arises entirely from general reflex movements, and that the action of the strychnine not only excites the motor fibres, but goes to increase the necessary cause from which reflex movements arise. If this be true, tetanus must cease, even when the spinal marrow is uninjured, provided the conditions under which reflex movements arise are removed. In order to test this, the following experiments were instituted :—

Exp. XI. The skin of a frog was rubbed over with strong prussic acid (30 per cent.,) so that the superficial peripheral extremities of the nerves were paralyzed. Strychnine was exhibited ; no tetanus ensued, except when the animal was strongly shaken (thrown about, for example.)

Exp. XII. After laying open the spine of a frog, the posterior nervous roots were divided. Same result as above, or as when the posterior column of the cord was irritated with a needle.

Exp. XIII. The same experiment was repeated in a frog in which tetanus was present ; it ceased immediately on division of the posterior roots, and only recurred under the conditions first stated.

From these experiments, then, it may be concluded, that tetanus arising from the action of strychnine originates entirely and alone from reflex movements produced by the strychnine exciting to increased action the primary cause of the reflex motions themselves.

The next question that occurs is, what is the primary cause of these reflex movements? Here, however, we find ourselves in the field of physiological controversy ; as far as regards the question first considered, it is a matter of little moment how the controversy may be decided. It may be remarked, however, that tetanus induced by strychnine cannot be attributed to a general increased excitement of the nervous system. Were this the case, stronger contractions of the muscles dependent on the anterior cord laid bare in Exp. VIII. IX. and X. should have occurred rather before the administration of the poison than after it ; but this was not the case. Other experiments, which require to be repeated, however, seem to show that the cause of reflex motion is to be sought for in the grey matter of the spinal cord, that the strychnine acts, therefore, by exciting to activity this grey matter, or rather the ganglionic in masses composed of it. These experiments are the following :—

Exp. XIV. A needle was carefully passed down through the centre of the spinal marrow, of a beheaded frog, as far as the point where the nerves to the anterior extremities are given off, and then withdrawn. Strychnine was then administered. Tetanus occurred in the posterior extremities only.

Exp. XV. After laying open the spinal canal, the grey matter of the posterior half of the cord was destroyed as far as it gave off nerves to the posterior extremities. The movements of the latter were little affected by the operation; but tetanus did not ensue after the exhibition of strychnine.—*Ibid, from Ibid.*

